# Cytometric patterns reveal growth states of *Shewanella putrefaciens*

**DOI:** 10.1111/1751-7915.12154

**Published:** 2014-09-03

**Authors:** Susanne Melzer, Gudrun Winter, Kathrin Jäger, Thomas Hübschmann, Gerd Hause, Frank Syrowatka, Hauke Harms, Attila Tárnok, Susann Müller

**Affiliations:** 1 LIFE – Leipzig Research Center for Civilization Diseases, University of LeipzigLeipzig, Germany; 2 Department of Pediatric Cardiology, Heart Center Leipzig, University of LeipzigLeipzig, Germany; 3 Interdisciplinary Center for Clinical Research, Core Unit Fluorescence-Technology, University of LeipzigLeipzig, Germany; 4 Translational Center for Regenerative Medicine, University of LeipzigLeipzig, Germany; 5 Department of Environmental Microbiology, Helmholtz Center for Environmental ResearchLeipzig, Germany; 6 Department of Biology: Physiology and Biochemistry of Plants, University of KonstanzKonstanz, Germany; 7 Biocenter, Martin-Luther University Halle-WittenbergHalle-Wittenberg, Germany; 8 Interdisciplinary Center for Material Sciences, Martin-Luther University Halle-WittenbergHalle-Wittenberg, Germany

## Abstract

Bacterial growth is often difficult to estimate beyond classical cultivation approaches. Low cell numbers, particles or coloured and dense media may disturb reliable growth assessment. Further difficulties appear when cells are attached to surfaces and detachment is incomplete.

Therefore, flow cytometry was tested and used for analysis of bacterial growth on the single-cell level. *Shewanella putrefaciens* was cultivated as a model organism in planktonic or biofilm culture. Materials of smooth and rough surfaces were used for biofilm cultivation. Both aerobic and anaerobic as well as feast and famine conditions were applied. Visualization of growth was also done using Environmental Scanning and Phase Contrast Microscopy. Bioinformatic tools were applied for data interpretation.

Cytometric proliferation patterns based on distributions of DNA contents per cell corresponded distinctly to the various lifestyles, electron acceptors and substrates tested. Therefore, cell cycling profiles of *S. putrefaciens* were found to mirror growth conditions.

The cytometric patterns were consistently detectable with exception of some biofilm types whose resolution remained challenging. Corresponding heat maps proved to be useful for clear visualization of growth behaviour under all tested conditions. Therefore, flow cytometry in combination with bioinformatic tools proved to be powerful means to determine various growth states of *S. putrefaciens*, even in constrained environments. The approach is universal and will also be applicable for other bacterial species.

## Introduction

Bacteria are unicellular organisms that live either planktonically or as biofilms in nearly all environments on earth, and they comprise essential functions in all respects (Houry *et al*., 2012; Ishihama, 2012; Rizoulis *et al*., 2013). Performances of bacteria in nature or in biotechnological processes correlate with abundances of cells and, thereby, their ability to grow. However, accurate analysis of bacterial growth can be challenging. Apart from using microscopic methods, quantification of bacterial growth is commonly done by biomass analysis. This classical approach has limitations, especially if media are opaque or biofilms are firmly attached to surfaces. Another problem is the enrichment of matrix particles by biomass harvest from porous surfaces resulting in error-prone biomass estimation by dry weight (DW) or optical density (OD) measurements (Uría *et al*., 2011). The biomass analysis can also be strongly influenced by changes in the cell's morphology-like variations in granularity and cell size. Another typical problem is the accumulation of storage products that increase biomass values although no growth is ongoing. An alternative and promising method to study bacterial growth is the analysis of DNA pattern using flow cytometry (FCM), which is tested in this study.

Deoxyribonucleic acid patterns provide information on cell proliferation activity. Proliferation of bacterial cells can be followed by quantification of chromosome numbers per cell (Müller, 2007). Replication of chromosomes is the most prominent sign for growth of a cell. Flow cytometry uses the fact that the chromosome numbers per cell depend on proliferation activity. The bacterial cell cycle is subdivided into three periods: the time between the ‘birth’ and initiation of replication (usually one chromosome equivalent, C_1n_), DNA replication (with two chromosome equivalents at the end of the phase, C_2n_) and cell division (C_1n_ again).

Flow cytometry measures thousands of single cells within few seconds. When measuring cellular DNA contents, the first sub-population of a histogram contains cells with one single chromosome equivalent, the C_1n_ cells. The second sub-population [when having the double mean fluorescence intensity (MFI)] contains cells with the double chromosome equivalent, the C_2n_ cells. In between are proliferating cells. But the bacterial cell cycle can be more complex (Müller and Nebe-von-Caron, 2010; Müller *et al*., 2010). The most widely studied bacterium in this respect is *Escherichia coli*. A special feature of the *E. coli* cell cycle is the uncoupled DNA synthesis: at optimal growth, new replication rounds are initiated in preceding generations (Cooper, 2006). In the DNA histogram, therefore, bacterial cells with multiple copies of the chromosome can be found for a fast-growing culture. Cell division usually occurs symmetrically and is defined as a dynamic event during which a cell divides into two equal cells. But this does not apply for all bacteria. The *α-Proteobacterium Caulobacter crescentus* for instance divides asymmetrically producing two morphologically and functionally different cells (Tsokos and Laub, 2012). Asymmetric division also occurs in a way that chromosome numbers are distributed unequally. In this case, the distribution of chromosomes is not log2 but linearly scaled. An example is *Desulfobacula toluolica* grown on toluene under sulfate-reducing conditions (Vogt *et al*., 2005). Bacteria can also contain much more than one or two chromosomes. An overview is given by Müller (2007).

We used *S. putrefaciens* in this study to relate growth dynamics to particular micro-environmental conditions. The strain can grow in planktonic (Beliaev *et al*., 2001) or biofilm lifestyles (Carmona-Martínez *et al*., 2013) and under aerobic or anaerobic conditions (Martín-Gil *et al*., 2004). The organism belongs to the *α*-*Proteobacteria*, is widely distributed in the environment in aquatic habitats and soils (Long and Hammer, 1941) and known to be a relevant human pathogen (Vogel *et al*., 1997). It is, besides using oxygen, capable of using various anaerobic electron acceptors like nitrate, nitrite, fumarate, thiosulfate and Mn(III) or Fe(III) oxides (Saffarini *et al*., 1994). Furthermore, the strain is known as an electricity-generating organism using electrodes as electron acceptors (Borole *et al*., 2011).

In this study, we aim for accurate analysis of cell growth in microbial samples that cannot be fully recovered from the environment without cell loss (e.g. biofilms), contain high amounts of particles or are cultivated in coloured media. To reach this aim, *S. putrefaciens* was used as model organism due to its versatile metabolism and lifestyle. The organism was cultivated under feast and famine conditions where we expected different proliferation activities. We compared classical methods for growth measurement with FCM and developed a workflow and a visualization procedure for population growth dynamics on the basis of single-cell analysis. Variation in growth behaviour under altering micro-environmental conditions was assessed by similarity analysis using bioinformatic tools.

## Results

### Classical growth analyses

**Fig 1:**
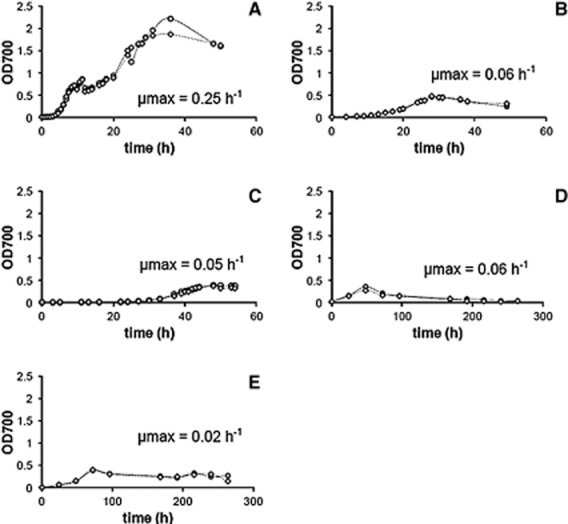
Growth curves of *S. putrefaciens* cells in planktonic (A–D) and biofilm cultures (E). Bacterial growth was measured by increase in OD at 700 nm. Growth rates (μmax) were calculated from the steepest rise of the growth curves. Planktonically grown cells were cultivated on peptone (feast/aerobic growth, A), lactate (famine/aerobic growth, B and D) and lactate/ferric–citrate (famine/anaerobic growth, C) in duplicates. Cultivation on lactate in the presence of glass wool was shown for planktonic cells in (D) and biofilm cells in (E).

Detection of growth in planktonic cultures: *S. putrefaciens* was grown both aerobically and anaerobically as planktonic cultures on peptone and lactate respectively (Fig. 1). Planktonic cultivation was chosen because of the relatively fast growth of cells and their simple harvest directly from the cultivation medium. Fastest growth of *S. putrefaciens* (μmax = 0.25 h^−1^; Table 1; Fig. 1A) was achieved by aerobic cultivation on peptone, measured at OD 700 nm. Growth on lactate under aerobic and anaerobic conditions was slower with μmax = 0.06 h^−1^ and μmax = 0.05 h^−1^ respectively (Table 1; Fig. 1B and C). Lactate was metabolized during 30–50 h of cultivation (Fig. S1). Ferric citrate was used as electron acceptor under anaerobic conditions and caused a colour change of the medium from yellow to green. In addition, aggregates of iron-complex-compounds precipitated, impeding an accurate analysis of growth activity by OD measurement.

**Table 1:** Overview of different cultivation experiments to analyse different lifestyles of *Shewanella putrefaciens*

							Microscopy					
Cultivation technique	Incubation method	C-source	Aerobic/Anaerobic		Growth condition	Maximal growth rate	PC	Fl	3D	ESEM	FCSM	Dalmatian Plot
Batch	Shaking flask	Peptone	X	–	feast	0.25 h^−1^	X	X	n.d.	n.d.	X	X
Batch	Shaking flask	Lactate	X	–	famine	0.06 h^−1^	X	X	n.d.	n.d.	X	X
Batch	Shaking flask	Lactate + iron(III)citrate	–	X	famine	0.05 h^−1^	X	X	n.d.	n.d.	X	X
Flow through chamber	FTC with GS	Lactate	X	–	famine	n.d.	X	X	X	n.d.	X	X
Batch	Shaking flask with GW	Lactate	X	–	famine	0.06 h^−1(plankt)^	X	n.d.	n.d.	n.d.	X	X
			X	–	famine	0.02 h^−1(biofilm)^	X	n.d.	n.d.	X	X	X
Static chamber	Static chamber with GP	Lactate	–	X	famine	n.d.	n.d.	n.d.	n.d.	X	X	X

C-source, carbon and energy source; PC, phase contrast microscopy; Fl, fluorescence microscopy; GS, glass slide; GW, glass wool, GP, graphite paper; n.d., not determined; X, was done in this study.

**Fig 2:**
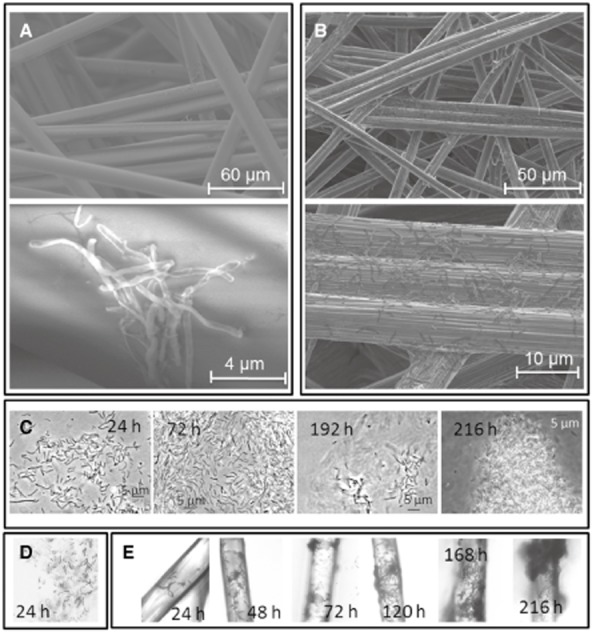
Microscopical analysis of bacterial growth on different surfaces. Environmental scanning electron microscopy imaging of a 24 h old biofilm on glass wool (A) and a 240 h old biofilm on graphite paper (B) in different magnifications. Single rod-shaped cells cluster as initial structures on the glass wool surface. Cells were distributed with higher densities on the graphite fibre surface. Growth of biofilms on glass slides during 24 h to 216 h was analysed by phase contrast microscopy (C). During the first 24 h of growth cells attached in one layer on the glass surface, after 72 h multilayered stacks were found, resulting in mushroom-like 3D structures after 240 h cultivation. (D) Planktonically grown cells from the glass wool growth approach after 24 h incubation. (E) Biofilm growth on glass wool during 24 h to 216 h. Attachment of single cells (24 h) was observed, resulting in dense growth on area spots onto the total glass wool (72 h, 120 h, 168 h) and mushroom-like structures (216 h), as observed for growth on the glass slides.

Detection of growth in biofilms: *S. putrefaciens* was grown both aerobically and anaerobically on lactate on two different glass surfaces and graphite paper. The specific growth parameters are shown in Table 1. The glass slides had a relatively smooth surface, whereas the glass wool had a rougher surface and an artificial 3D structure of grooves and micro-niches (Fig. 2A). The biomass harvest was similar with 1.6 mg cm^−2^ for glass wool and 1.3 mg cm^−2^ for glass slides after 216 h. In addition to biofilm growth, the conspecific planktonic populations were also studied. In the glass wool experiment, growth rates were found of μ = 0.06 h^−1^ and μ = 0.02 h^−1^ for planktonic and biofilm grown cells respectively (Fig. 1D and E; Table 1). Small spots of the glass fibres were covered by biofilms already after 24 h incubation, as shown by environmental scanning electron microscopy (ESEM) (Fig. 2A) and phase contrast microscopy (PC; Fig. 2E). Despite strong agitation, biofilms developed into spot-like 3D structures over the next 216 h. A similar colonization behaviour was found for the static glass slide cultures (Fig. 2C).

Biomass of cells grown on graphite paper could not be estimated. Mechanical cell detachment was difficult due to the cells' growth inside the porous structure of the rough surface. Therefore, biomass was low, and the sample preparations were rich in matrix particles. Cell attachment on graphite paper was imaged by ESEM after 240 h cultivation (Fig. 2B). The arrangement of the graphite paper fibres was comparable to that of the glass fibres, and dense biofilms grew until the end of the experiment (336 h). Nevertheless, less than 6 × 10^6^ cells cm^−2^ could be detached from the graphite paper at this time point.

### Detection of growth using FCM

The measurement of DNA patterns of single cells by FCM served to determine proliferation activities of cells. They are expected to mirror the growth rate of a population and be helpful in estimating growth activity if other techniques like DW or OD determination fail. In this study, we used FCM to analyse *S. putrefaciens* when grown in planktonic or biofilm lifestyles on different surfaces, various substrates and electron acceptors. Samples from all cultivation approaches were taken regularly, analysed by FCM, and the abundances of cells with respective chromosome equivalents were determined.

**Fig 3:**
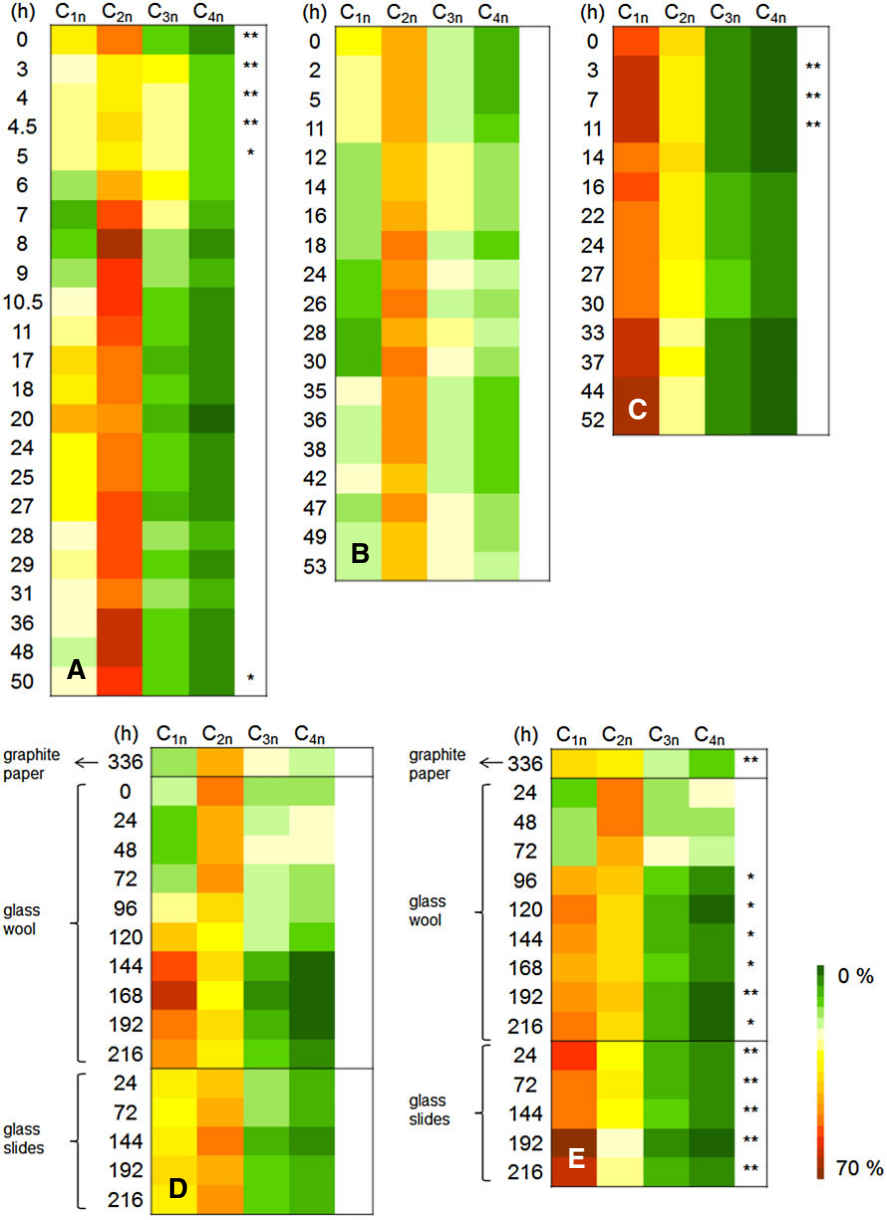
Growth patterns of planktonic and biofilm cells of *S. putrefaciens*. Deoxyribonucleic acid distribution pattern of nine different growth conditions were compared in a heat map with each other. Distribution of DAPI stained cells with C_1n_, C_2n_, C_3n_ and C_4n_ chromosome contents were measured by FCM over cultivation time when planktonically grown under feast/ aerobic (A), famine/ aerobic (B), famine/ anaerobic (C) and in matrix containing approaches (D). Data of biofilm grown cells are shown in (E), corresponding to the planktonic cells of D. Time of cultivation is given in the first row of each heat map. The colour key marks abundance of cells in the C_1n_-C_4n_ sub-populations from 0–70 % (colour key) of DAPI-stained cells. Possibly emerging unstained cells are highlighted by one (frequency > 10%) and two stars (frequency > 25%) in the last column of each table. The results rely on one-parametric analysis of DAPI histograms. In depth analysis of two dimensions (scatter versus DAPI fluorescence) revealed dynamics in bacterial growth (Figs 6). Fig. S4 shows the abundances of all cells.

Four sub-populations with distinct DNA contents were clearly distinguished (Fig. 3). Geometric MFI values of 4'6-diamidino-2-phenylindole (DAPI) channel numbers were calculated for all samples to be 98.0 (C_1n_), 183.0 (C_2n_), 282.5 (C_3n_) and 388.7 (C_4n_), as shown in Fig. S2. Some samples showed more sub-populations with even higher DNA contents. Due to cell cycling events, cells virtually shifted between the DNA distributions over time, and their varying number per distribution was evaluated. The outcome is visualized in a coloured heat map (Fig. 3) by using a normalization approach (Fig. S3), enabling the comparison of all experiments (Fig. S4). The heat map in Fig. 3 shows cytometric DNA sub-population distribution over time containing cells with up to four chromosomes (C_1n_–C_4n_). The cell abundances at 0 h mirrored the proliferation state of the cells when inoculated. While, e.g. the inoculum for the complex peptone medium (Fig. 3A) originated from the exponential growth phase of a pre-culture, the inoculum for the anaerobic cultivation on minimal medium (Fig. 3C) was drawn from a respective stationary pre-culture.

In this study, two types of pattern distributions were observed for *S. putrefaciens*.

The first type envisages major parts of the cells in the C_2n_ distribution. Planktonic growth caused high abundances of cells in the C_2n_ distribution (Fig. 3A and B). When *S. putrefaciens* was grown on a complex carbon and energy source like peptone (Fig. S5), or under aerobic conditions on lactate (Fig. S6), a major part of cells maintained double chromosome contents during the whole cultivation time. Such behaviour was also observed for planktonic cells in the flow-through chamber (FTC; Fig. S7) and for the glass wool experiments (Fig. S8) during the first days of cultivation. Even in the graphite paper chamber, cells (Fig. S9) preferred the C_2n_ instead of the C_1n_ state, suggesting that growth was rather fast under such conditions (Fig. 3D). *Shewanella putrefaciens* showed even higher growth velocities for only 2 h on peptone in the early exponential growth phase. Here, the organism generated a uniform distribution of cells between sub-populations C_1n_ to C_4n_ (Fig. 3 and Fig. S4, 3–5 h), and is undergoing probably uncoupled DNA synthesis for a short-time interval.

The second type envisages major parts of the cells in the C_1n_ distribution. Under anaerobic growth conditions (Fig. S10), the highest abundances of cells were found in the C_1n_ distributions in all samples (Fig. 3C and D). The carbon source lactate caused obviously limiting proliferation activity for *S. putrefaciens* when oxygen was not available as electron acceptor. In addition, all biofilm samples showed accumulation of cells in C_1n_ (Fig. 3E), with exception of early biofilms on glass wool (Fig. 3E, 24–72 h). However, also the planktonic cells in the aerobically grown culture that contained glass fibres as a compact attachment matrix accumulated in C_1n_ after a longer cultivation time (Fig. 3D, 120–216 h). Besides the cells' distribution in C_1n_ to C_4n_ subsets, some of the cells remained unstained or showed cells with lower stainability (Fig. S8). High proportions of unstained cells were found in nearly all biofilm samples and, surprisingly, also in the first hours during cultivation under feast growth conditions (Fig. S5).

### Comparison of population dynamics using the Dalmatian tool

To better describe and compare population behaviour of *S. putrefaciens* under diverse growth conditions, cell side-scatter characteristics (SSC) were followed in addition to chromosome numbers per cell. The two parameter FCM analysis created 2D plots and allowed a defined sub-population distinction. The workflow is the following: The 2D plots mirrored subsets of cells. Depending on growth conditions, theses subsets appeared or disappeared by changing the number and position of sub-populations in the 2D plot. These variations can be evaluated by using the Dalmatian tool (Bombach *et al*., 2011). The tool compares 2D plots which each other and follows trends in population behaviour (Müller *et al*., 2012). Side-scatter characteristics : deoxyribonucleic acid sub-populations were defined for each 2D plot by gate settings, position corrected if required (Fig. S11), and the Dalmatian plot evaluation performed as described in *Experimental procedures*. Results allowed trend interpretations and similarity estimation in population dynamics.

**Fig 4:**
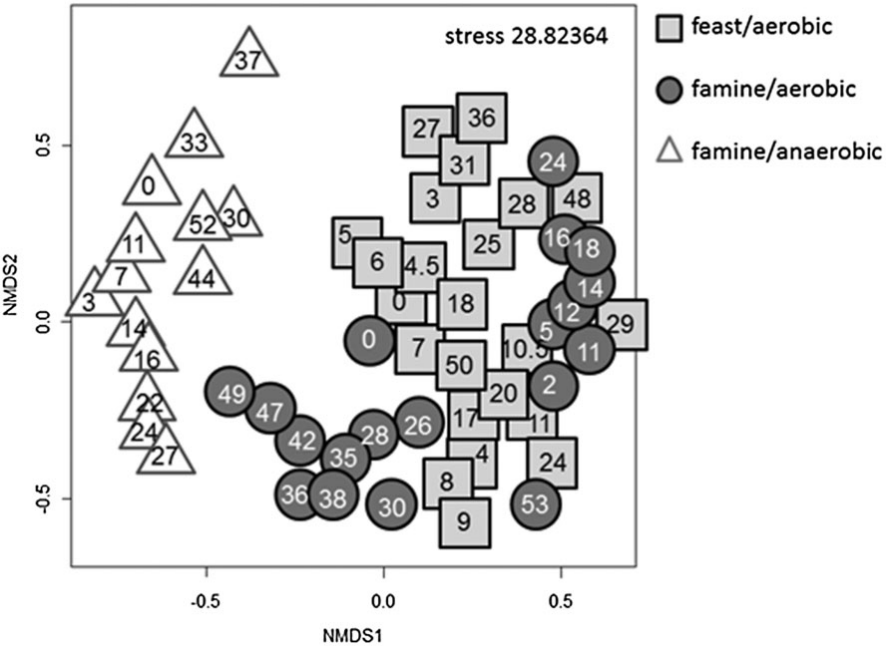
Similarity analysis of SSC : DNA patterns of planktonic cells under feast/famine growth conditions. Dalmatian black and white SSC : DAPI plots of three different batch cultivation approaches are shown. In detail, planktonic cells on feast/aerobic (cube), famine/aerobic (circle) and famine/anaerobic (triangle) growth conditions were compared. Numbers within the geometric figure symbol the time points of the sample in hours.

The nMDS plot in Fig. 4 clearly showed similarities or dissimilarities in the proliferation activity of *S. putrefaciens* over time due to the different cultivation conditions. A clear dissimilarity was observed for the cells' proliferation behaviour under aerobic and anaerobic conditions. In addition, cells that had access to oxygen but were cultivated under famine or feast carbon conditions were distinguishable as well in the nMDS plot, although they clustered in near vicinity. Hence, the nMDS plot reflected the proliferation pattern shown in Fig. 3A and B where aerobically grown cells accumulated a major part of the cells in the C_2n_ distribution, while anaerobically grown cells stayed longer in C_1n_ (Fig. 3C).

**Fig 5:**
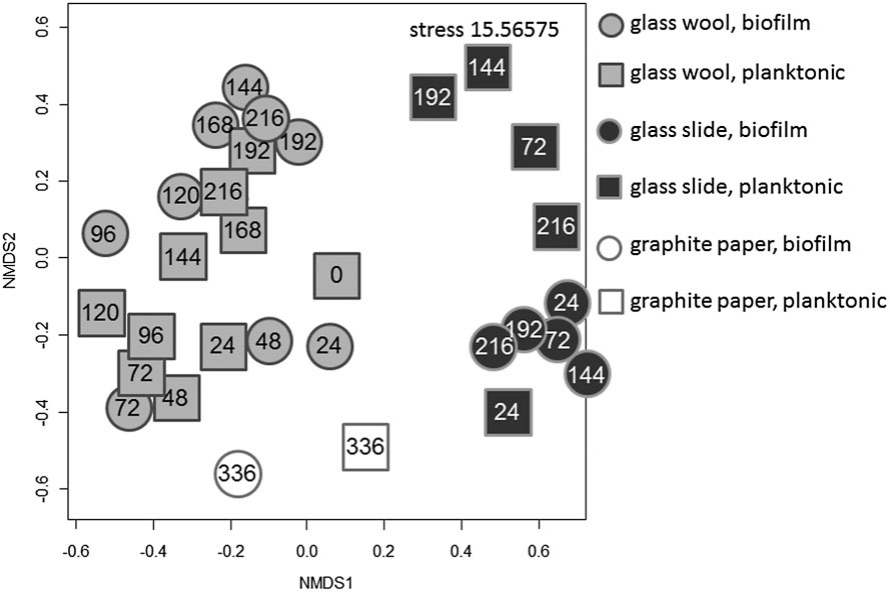
Similarity analysis of SSC : DNA patterns of biofilm and planktonically grown cells. Dalmatian black and white SSC : DAPI plots are shown from biofilm grown cells on glass wool, glass slides and graphite paper as well as from corresponding planktonically grown cells. Aerobic cells from the glass wool approach in batch culture (biofilm: light-grey filled circle; planktonic cells: light-grey filled cube) were compared with aerobic biofilm (dark grey filled circle) and planktonic (dark grey filled cube) cells from the FTC experiment and with anaerobic biofilm cells from the graphite paper (unfilled circle plus the corresponding planktonic cells, unfilled cube).

Furthermore, the Dalmatian tool was used to show dissimilarities in the cells' proliferation behaviour when cells were grown in planktonic or biofilm lifestyles (Fig. 5). Glass slide, glass wool and graphite paper biofilms clustered distinctly, due to their different SSC : DNA characteristics (Fig. 5, circles). Plainly, the planktonic cells thriving in the identical environments were found in the vicinity of the respective biofilm clusters for all three approaches (Fig. 5, squares). The Dalmatian plot findings suggest that planktonic and biofilm cells show similar growth characteristics in identical environments. Such a behaviour is also reflected in Fig. 3D and E, although abundances of cells in sub-populations are better reflected in the heat map.

**Fig 6:**
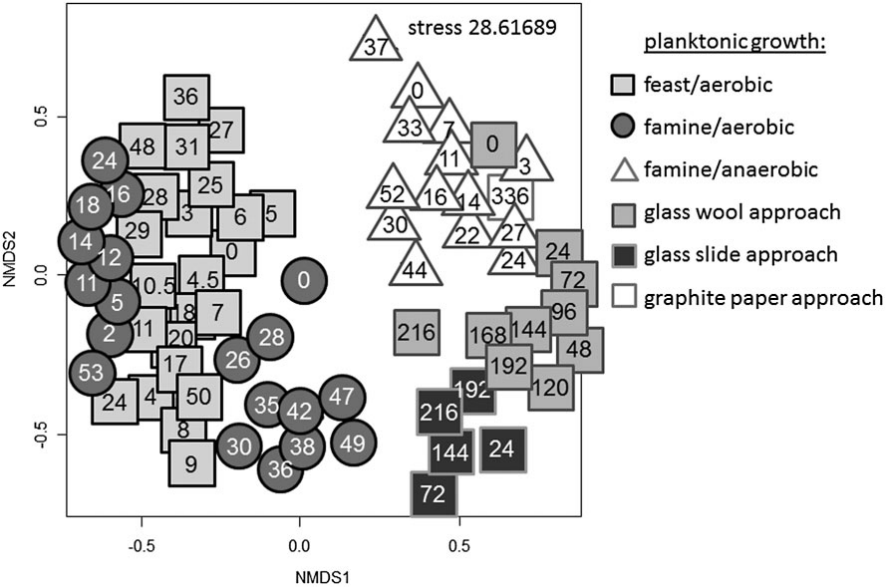
Similarity analysis of SSC : DNA pattern of all planktonic cells. Dalmatian black and white SSC : DAPI plots are shown of all planktonic cells of batch cultures and the matrix containing growth approaches. The same symbols were used as in Figs 4 and 5.

As the proliferation activity of the planktonic cells in the aerobic glass wool approach was surprisingly low in FCM (Fig. 3D) in contrast to OD analysis (Table 1), the nMDS plot was used to compare these findings with all other planktonically grown samples. As expected, these planktonic cells (light-grey cubes) clustered evidently dissimilar from the cells cultivated without the solid glass fibre matrix (Fig. 6) but were, nevertheless, similar to the anaerobically grown cells (unfilled triangles) and even to planktonic cells from the FTC (filled squares).

The evaluation of the various DNA : SSC dot plot patterns of *S. putrefaciens* using the Dalmatian tool verified the data obtained by evaluation of single parameter DNA patterns and showed the multiple behaviour of the organism under the various growth conditions. Lifestyle, in addition to carbon source and electron acceptor availability, influenced the proliferation velocity of *S. putrefaciens*. Flow cytometry in combination with bioinformatic tools was perfectly able to document these variations even when classical techniques as analyses of DW or OD are likely to fail.

## Discussion

Microbial growth is usually quantified by measurement of biomass over time. Besides DW analyses, OD is most often used for the purpose. Other techniques are analyses of increasing contents of macromolecules like total protein (e.g. Stickland, 1951), ribosomal RNA contents (e.g. Moller *et al*., 1995) or other intrinsic cell markers (e.g. Unge *et al*., 1999). Increase in cell counts can be determined by microscopy, coulter counter techniques or FCM. All of them can be error prone, especially, if detachment of cells from surfaces is difficult or particles are present.

We aimed to determine growth states of *S. putrefaciens* by measurement of chromosome numbers in individual cells (Müller, 2007; Müller and Nebe-von-Caron, 2010; Müller *et al*., 2010). The advantage of this approach is that no further knowledge of biomass quantities or cell number is necessary. Already an amount of about 20 000 cells is sufficient to create a highly resolved cytometric dot plot. Complete recovery of cells from the growth matrix is not required. Neither coloured or opaque media (Neumeyer *et al*., 2013) nor lower than equi-proportional particle numbers disturb such analyses because they do not specifically bind the used DNA specific fluorescent dye.

When applicable, classical analysis of biomass was used in this study to quantify growth of *S. putrefaciens* in comparison to FCM. Generally, when OD measurement was possible, the FCM data mirrored growth behaviour under the respective conditions. However, biofilm samples did not allow continuous sampling for biomass detection due partly to low cell density and tight attachment to surfaces. Further, following biofilm cell growth by microscopic techniques was elaborate. In addition, quantitative evaluation of biofilm mass would require specific software tools which are, to our knowledge, still not applicable. Therefore, only limited and flawed classical biomass information was available for the biofilm grown cells but also for some of the anaerobically grown samples.

In contrast, high-resolution FCM and the highly DNA-specific dye DAPI were useful for quantitative growth determination in all tested samples. The method gave cell cycle and growth behaviour information on the strain that was not available before.

In general, *S. putrefaciens* was surprisingly found to contain at least four chromosome equivalents and divide in an asymmetric way. Apart from symmetric cell division (C_2n_) with sub-populations containing 1, 2 or 4 chromosome equivalents, a sub-population comprising a C_3n_ chromosome amount was detected. Additionally, C_5n_ states, also characteristic for asymmetric cell division, could often be distinguished in samples grown under famine conditions (Fig. S4). However, because cells in the C_5n_ states were of low frequency, they were disregarded for the overview presentation of proliferation states in Fig. 3 (like all other additional sub-populations, see below). Nevertheless, extended heat maps contain abundance variation within all subsets of cells to enable growth velocity evaluation, as shown in Fig. S4. This figure provides even higher resolved information on proliferation states of the strain under the various conditions. An evaluation of growth states was possible by comparing the varying numbers of cells in the upcoming sub-populations using bioinformatics tools. Heat maps (Fig. 3; Fig. S4) and Dalmatian plots (Müller *et al*., 2012) were created to facilitate the overview on the variations and found to mirror the various growth states of *S. putrefaciens* like fingerprints. Both tools showed similar outcomes although based on distinct bioinformatics evaluation principles and even independent cell-based information.

Fastest growth was found on aerobic peptone medium, a complex mixture of peptides and amino acids as carbon source. Here, even uncoupled DNA synthesis was observed for the time range of 3–5 h, a behaviour that was described in detail for *E. coli* under similar growth conditions (Cooper, 2006). When lactate was used as carbon source, the proliferation activity decreased. The slower growth was caused by the low carbon source quantity in contrast to the energy-rich peptide mixture. A further decrease in proliferation activity was found when cells were grown aerobically on lactate but in the presence of glass wool. Here, the population also preferred the C_1n_ state after about 4 days of cultivation. It can only be speculated that the glass wool represent an interfering component during the aerobic cultivation approach. Shear stress on the glass fibres addressed the cells from all directions and might be the reason for the slow growth in these shake flasks experiments. Cells might need additional energy to repair damages occurring in the process.

Shewanella strains are known to oxidize lactate completely under aerobic conditions but can also use it under anaerobic conditions, here in the presence of iron(III)citrate. Due to this behaviour, the organism is often enriched at oxic/anoxic interfaces of aquatic habitats (Scott and Nealson, 1994). Under these conditions, very low proliferation activity was analysed. When substrate is limiting and electron acceptors have a low-reducing potential bacteria are known to undergo maintenance states (Russell and Cook, 1995). The reduced growth conditions obviously resulted in the accumulation of cells in the C_1n_ state.

The study of *S. putrefaciens* is of importance, as the organism might be useful to convert chemical energy into electrical energy but also vice versa to produce bulk chemicals in an environmental friendly way (Wang and Ren, 2013). Some efforts to follow growth characteristics of *S. putrefaciens* have been made recently. Franks and colleagues (2008) developed a real-time three-dimensional imaging system to visualize live, active microbial biofilms in microbial fuel cells with confocal scanning laser microscopy. However, biofilms of *S. putrefaciens* are described to be often tenuously distributed on surfaces (Bagge *et al*., 2001), which was also observed in this study. In contrast, growth of another electricity generating organism, an engineered mCherry-labelled *Geobacter sulfurreducens* strain, was described to grow more compact, but accurate quantification of growth remained still difficult (Franks *et al*., 2009). A first attempt to use FCM for the purpose was already made (Harnisch *et al*., 2011) and shows the potential of the method on *G. sulfurreducens*. In this study, *S. putrefaciens* was grown on graphite paper using the cultivation assembly described in the Supporting Information. In contrast to the former study, the detection of the proliferation pattern remained limited. A huge part of the cells could not be stained requiring further developmental steps of cell handling in future.

It should not remain undiscussed that high numbers of unstained cells were found in several experiments. It is known from other approaches that stressed cells with a dense cell wall withstand staining with fluorescent dyes (Müller and Nebe-von-Caron, 2010). Obviously, larger parts of cells in biofilms or in populations that were freshly inoculated into new media protect themselves against harsh or drastically changing conditions by increasing the density of the cell wall. This behaviour is often observed for medically relevant strains (Mah and O'Toole, 2001; Cui *et al*., 2003). In addition to this technical phenomenon, we also would like to point to the so called ‘ghost cells’ found in natural environments without nucleic acid contents (Zweifel and Hagstrom, 1995). Nevertheless, to decrease the proportion of unstained cells, the cell handling and staining procedures should be further improved. Nonetheless, the rest of the population gave a clear indication of sub-populations diverging towards different chromosome contents.

In general, the states and features of cells over time (proliferating, highly proliferating, non-proliferating, stained/less stained/unstained) and their variation in dependence of abiotic factors generate huge data sets that make up a so-called cytome. In its original definition, a cytome is defined as the entity of all cells in an organ that are interrelated and connected (Valet *et al*., 2004), but also bacterial populations and communities are known to act in a concerted manner if required. Following behaviour and dynamics of whole bacterial cytomes may open another way to a better understanding of bacterial population performances under varying growth conditions (Koch *et al*., 2014). The presented workflow for determining growth states of *S. putrefaciens* under varying conditions is universal. It simply requires sampling of at least 20 000 cells, fixation, staining of the DNA, FCM and bioinformatic approaches for cytometric data evaluation. But other bacteria will ultimately show a different growth behaviour, therefore, reference proliferation patterns will always be required.

## Conclusion

The study aimed to describe means for determination of cell growth and proliferation when classical approaches like DW, OD or cell count detections fail. Here, we tested biofilm versus planktonic growth, representing two main different lifestyles of bacteria. In addition, different growth velocities were investigated by variation in substrates (feast versus famine) and electron acceptors (e.g. oxygen versus iron(III)citrate). Flow cytometry and bioinformatics were used for sample analysis and evaluation tools.

The proliferation patterns of *S. putrefaciens* were directly influenced by the substrate source, the used electron acceptor and planktonic or sessile lifestyles. As a main trend, slow growth was accompanied by an accumulation of cells within the C_1n_ state, while faster growth prompted cells into the C_2n_ state. This knowledge may be useful for stimulation and optimization of various biotechnological processes, where microbial biofilms are often the main producers. Comparison of unknown cytometric patterns with well-known ones allows estimating the proliferation activity and, as a consequence, growth. It has thus the potential for trend interpretation and prediction of population behaviour and selective stimulation of bio-processes. In addition, correlation analyses of FCM data with other ‘abiotic’ parameters will further highlight potentials or shortcomings in bioprocesses in future.

## Experimental procedures

### Materials

All chemicals were, unless otherwise specified, obtained from Merck KGaA (Darmstadt, Germany), Sigma-Aldrich (St. Louis, MO, USA), Hoffmann-La Roche AG (Basel, Switzerland) and Carl Roth GmbH + Co (Karlsruhe, Germany), with at least technical grade.

The lactate minimal medium (pH 7.4) was used for cultivation of (I) aerobic planktonic cells in batch culture, (II) aerobic biofilms on glass wool, (III) aerobic biofilms on glass slides in a flow-through chamber and (IV) anaerobic biofilms on graphite paper. Its composition was as followed: lactate 2.67 g l^−1^, NH_4_Cl 0.95 g l^−1^, K_2_HPO_4_ × 3 H_2_O 1.301 g l^−1^, KH_2_PO_4_ 0.449 g l^−1^, NaHCO_3_ 0.168 g l^−1^, L-arginine 0.02 g l^−1^, L-glutamate 0.02 g l^−1^, L-serine 0.02 g l^−1^, CaCl_2_ × 2 H_2_O 0.057 g l^−1^, CoSO_4_ × 7 H_2_O 1.41 mg l^−1^, CuSO_4_ × 5 H_2_O 0.042 mg l^−1^, ethylenediaminetetraacetic acid (EDTA) 0.02 g l^−1^, FeSO_4_ × 7 H_2_O 1.7 mg l^−1^, H_3_BO_3_ 2.8 mg l^−1^, MgSO_4_ × 7 H_2_O 0.2 g l^−1^, MnSO_4_ × H_2_O 0.17 mg l^−1^, (NH_4_)_2_SO_4_ 0.66 mg l^−1^, Na_2_MoO_4_ × 2 H_2_O 0.75 mg l^−1^, Na_2_SeO_4_ 0.03 mg l^−1^, Na_2_SO4 0.7 mg l^−1^, NiCl_2_ × 6 H_2_O 1.365 mg l^−1^, ZnSO_4_ × 7 H_2_O 0.24 mg l^−1^. For cultivation of anaerobic planktonic cells in batch culture, lactate-Fe(III)citrate medium (pH 7.0) was used. The composition of the medium was equal to the lactate medium but was supplemented with Fe(III)citrate 2.5 g l^−1^ and lacked CaCl_2_ × 2 H_2_O and EDTA. For growth on complex medium DSMZ No 948 (pH 7.0) was used.

*Shewanella putrefaciens* 6067^T^ was purchased from DSMZ (Leibniz Institute, German Collection of Microorganisms and Cell Cultures, Braunschweig, Germany).

### Analysis of growth

Optical density was measured at OD_700nm_, d = 5 mm, using the Spectrophotometer UV/VIS Ultrospec III (Amersham Pharmacia Biotech, Piscataway, NJ, USA).

Dry weight was measured using 500 μl cell solution that was centrifuged at 3200*g for 20 min. The supernatant was carefully removed. Duplicates of each sample were dried overnight in a hot drying oven (80°C) and finally weighed.

Phase contrast microscopy was used to observe planktonic and biofilm samples on glass wool and glass slides (Axioskop, Type B, Zeiss, Germany). Pictures were taken with Sony Progressive 30 CD camera and analysed with Openlab 3.1.4 (Improvision, Lexington, MA, USA). A Plan-Neofluar 100×/1.30 objective was used with oil. Fluorescence was analysed after excitation by a 100 W mercury arc lamp (HBO W/2, 260 VA, Zeiss). Three-dimensional imaging was done using an Axio ObserverZ.1 (Carl Zeiss, Jena, Germany) with an LD LC1 Plan-Achromat 25×/63× objective to focus more deeply into the sample. Images were reordered on a digital camera (AxioCam MRm, Carl Zeiss).

Environmental Scanning Electron Microscopy (ESEM XL 30 FEG, FEI) served to observe biofilm growth on glass wool. Samples were analysed natively under low pressure as well as fixated samples under vacuum for higher resolution. Before microscopic analysis samples were fixated as follows: The material was fixed with 3% glutaraldehyde in sodium cacodylate buffer (SCB) pH 7.2 for 1 h at room temperature (RT), washed with SCB, postfixed with 1% osmiumtetroxide in SCB and finally washed with pure water. ESEM parameters for the single images were as followed: 5/8 kV gaseous secondary electron (GSE) detector, wet mode, 1.3 mbar (for glass-wool samples) and 2/5 kV, SE detector, Hi-Vac mode (for graphite anodes).

### Cultivation of *S. putrefaciens* as planktonic cells

*Shewanella* *putrefaciens* was grown in 500 ml flasks filled with 150 ml medium at 150 r.p.m. (shaking incubator TH25, SM-30 control, Edmund Bühler GmbH, Tübingen, Germany) and RT. While the cells were cultivated on peptone (5 g l^−1^ peptone) for feast growth, they were cultivated on aerobic lactate medium (lactate 2.67 g l^−1^, pH 7.4) and anaerobic lactate-Fe(III)citrate medium (lactate 2.67 g l^−1^, Fe(III)citrate 2.5 g l^−1^, pH 7.0) for famine growth. Under anaerobic conditions, cells were grown in 200 ml injection vials at RT and sealed with gas tight septa. The medium was degassed for 3 h with nitrogen. Cells were pre-cultivated on 0.5% peptone-agar plates for 14–16 h at 30°C. Single colonies were used for starter cultures and pre-cultivated in 25 ml peptone medium at 150 r.p.m. and 30°C. For inoculation of the main culture, 5–10 ml of the pre-culture were used to start with an optical density of 0.05. Growth was followed by measuring respective OD values. Cells were harvested and centrifuged at 3.200*g at RT for 10 min. The supernatant was transferred to a new tube to determine lactate concentration by ion chromatography (DX 100, Dionex, Sunnyvale, CA, USA). About 10^9^ cells were fixated with 1 ml fixation buffer [10 % sodium azide, 5 mM BaCl_2_ and 5 mM NiCl_2_ (Günther *et al*., 2008)]. Cells were stored at 4°C for up to 14 days. Each growth experiment was performed at least in biological duplicates.

### Cultivation of *S. putrefaciens* as biofilms

Glass slides, glass wool and graphite paper have different surfaces and were used as matrix for cultivation of *S. putrefaciens*. While the glass slide surface was smooth, those of glass wool was rougher. A real porous surface was provided by graphite paper. In this study, the attachment and growth of cells on these different surfaces was tested.

*Shewanella putrefaciens* was cultivated on glass wool in 150 ml lactate medium under aerobic conditions in 500 ml flasks filled with 2.5 g glass-wool (Merck chemicals, Cat. No. 104086, 15–25 μm in diameter with a total surface of approximately 3800 cm^2^/flask). The medium was inoculated as described before and cultivated for up to 216 h at 250 r.p.m. at RT. Biofilm samples were taken after 24 h, 48 h, 72 h, 96 h, 120 h, 144 h, 168 h, 192 h and 216 h. Also, planktonic samples (5–10 ml from the supernatant) were taken at these time points plus a sample from the inoculum. To harvest biofilm cells, victim flasks were used. For every sample time, the supernatant from one flask was discarded, and the complete glass wool was washed twice with 30 ml sterile PBS solution with addition of fixative (8 g l^−1^ NaCl, 0.2 g l^−1^ KH_2_PO_4_, 1.15 g l^−1^ Na_2_HPO_4_, 0.2 g l^−1^ KCl, 0.2 g l^−1^ NaN_3_, pH 7.2) for 10 min at 150 r.p.m. and RT. The supernatant was discarded, the glass wool transferred to a plastic shaking bottle and 10–15 ml PBS, and 45 g sterile glass beads (Sigma-Aldrich, G8722, 6 mm in diameter) were added. The wool was shaken at 400–450 r.p.m. for 30 min at RT. In a pre-test, samples were taken every 5 min for microscopic analysis of the detached cells. We found that incubation with the glass beads for 30 min was successful to fully detach all cells and to ensure that the cells' morphology was undisturbed. Optical density was measured as described above. For FCM, cells were transferred into a glass tube, centrifuged at 3200*g and RT for 10 min and fixated as described elsewhere.

*Shewanella putrefaciens* was cultivated on glass slides in an FTC under aerobic conditions (Fig. S12). A Hellendahl staining compartment (Carl Roth, 6 × 5.5 × 10.5 cm, volume: 350 ml) was used for the purpose. The chamber contained five glass slides at a size of 2.54 × 7.62 × 0.1 cm each and with a total surface of nearly 40 cm^2^ each (Carl Roth, Cat. No. 0656.1). The slides and the chamber were sterilized and inoculated with 1 ml starter culture and 150 ml lactate medium. With a flow rate of 4 ml h^−1^ (pump: Watson-Marlow 205 U) fresh medium was added to the compartment. Waste was removed continuously. Glass slide samples were taken after 24 h, 72 h, 144 h, 192 h and 216 h of cultivation for microscopy and FCM. Planktonic samples were taken at the same time points. For FCM sampling, slides were transferred to a 3 × 8 × 1 cm^3^ stainless steel chamber with 1.5 mm broad cavity. By sonification (ultrasonic bath USR-9, demand – P30/120 W, frequency f 35 kHz, Merck Eurolab NV) for 10 min, cells were detached from the glass surfaces. One hundred microlitres of the supernatant were used to determine DW as described before. The residual supernatant was transferred to a glass tube and centrifuged at 3200*g and RT for 10 min. Cells were fixated as described elsewhere. Growth conditions for cultivation of *S. putrefaciens* on graphite paper are described in detail in the Supporting Information section. All cultivation experiments are summarized in Table 1.

### DNA staining of cells and flow cytometry

To determine DNA distribution patterns, cells were stained with the AT-specific dye DAPI. Upon UV excitation, the DNA-bound dye fluoresces blue (λmax 461 nm) with a linear correlation between signal intensity and DNA content of the cell. With FCM, cells with different DNA contents were analysed, and distributions of cells with multiple DNA copies were represented in histograms. To stain DNA in *S. putrefaciens* fixated cells were centrifuged at 3200*g at RT for 10 min and re-suspended in PBS to an amount of 3 × 10^8^ cells per ml. Cells were washed twice with 2 ml PBS. Afterwards. cells were treated with 1 ml solution A (0.1 M citric acid in 0.5% Tween 20; v/v) for 30 min at RT. After centrifugation at 3.200*g at RT for 10 min, the cell pellet was re-suspended in 2 ml solution B (0.68 μM DAPI in 0.4 M Na_2_HPO_4_/NaH_2_PO_4_, pH 7.0) and stained for 1 h at RT in the dark. Flow cytometry analysis was carried out using a MoFlo cytometer (DakoCytomatation, Fort Collins, CO, USA) equipped with two water-cooled argon-ion lasers Innova 90 C and Innova 70C (Coherent, Santa Clara, CO, USA). Excitation at 488 nm (400 mW) was used for analysing the scatter signals. 4'6-Diamidino-2-phenylindole was excited by multi-line UV with 333–363 nm (100 mW). The orthogonal side scatter was first reflected by a beam splitter and then recorded after reflection by a 555 nm long-pass dichroic mirror; passage a 505 nm short-pass dichroic mirror and a band pass (BP) filter 488/10. DAPI fluorescence passed to a 450/65 BP filter prior to detection. Photomultiplier tubes were obtained from Hamamatsu Photonics (models R 928 and R 3896; Hamamatsu City). Amplification of signals was carried out at logarithmic scale, and the measurement of events was triggered by the SSC signal.

For system calibration and quality control cell counting, fluorescence beads (yellow-green fluorescent beads: 2 μm, FluoSpheres 505/515, F-8827, blue fluorescent beads: 1 μm, FluoSpheres 350/440, Molecular Probes Eugene, Oregon, USA) were used (Koch *et al*., 2013). An internal DAPI-stained bacterial cell standard was introduced for testing the sensitivity of the system by adjusting the coefficient of variation not higher than 5%.

### Analysis of flow cytometric data

Data were recorded and analysed using Summit software V 4.3 (DakoCytomation, Fort Collins, CO) and FlowJo V 7.6.4 (Tree Star, Ashland, OR) resulting in 2D plots of SSC versus DAPI fluorescence. Gating strategy and calculation of relative cell counts in per cent are described in Fig. S2. Quantitative information on DNA distributions were summed up in manually generated heat maps (Fig. 3, Fig. S4) for each growth experiment. Colours were defined by using the red-green-blue (RGB) colour space with values of 0–255: from dark green (30:100:0) to light green (200:250:50) in six steps with +20:+40:0 for step one to four and +50:+20:+75 for step five and six as well as light yellow (255:255:200) to dark red (140:50:0) in 14 steps. Following, the 2D plots were simplified into present/absent plots representing sub-populations as black coloured gates representing cells with certain SSC and DNA contents. With these so-called Dalmatian plots, similarity alignments were performed, resulting in Dalmatian nMDS plot. Within such plots, samples containing cells with similar features are grouped together, and samples with cells of different characteristics are found more distant (Bombach *et al*., 2011). For histogram comparison, pixel intensities and amounts were determined with ImageJ V 1.45. The resulting table with pixel values was used as a similarity matrix by an R-script (R-2.11.0, Development Core Team, 2009, R package vegan 1.17-1) to calculate nMDS similarity plots based on the modified Jaccard Index. To compare the various measurements over time, a normalization approach was performed. For this an intern bacterial standard was prepared, measured, and its changing relative MFI values were used to adjust the measurements on a daily base (Fig. S3). In addition, independence of plot position was tested for position-corrected versus non-position-corrected data comparison (Fig. S11).

## Conflict of interest

None declared.

## Supporting Information

**Fig. S1.** Substrate consumption of *S. putrefaciens*. **Fig. S2.** Gating strategy for differentiation of sub-populations with distinct DNA contents. **Fig. S3.** Instrument stability over time. **Fig. S4.** Dynamics in bacterial growth pattern of planktonic and biofilm cells. **Fig. S5.** DNA distribution patterns for feast growth under aerobic conditions. **Fig. S6.** DNA distribution patterns for famine growth under aerobic conditions. **Fig. S7.** DNA distribution pattern for planktonic and biofilm cells for the growth on glass slides. **Fig. S8.** DNA distribution pattern for planktonic and biofilm cells for the growth on glass wool. **Fig. S9.** DNA distribution pattern for planktonic and biofilm cells for growth on graphite paper. **Fig. S10.** DNA distribution pattern for famine growth under anaerobic conditions. **Fig. S11.** Dalmatian plot position tendency of comparative analysis via nMDS. **Fig. S12.** Schematic illustration of the flow-through-chamber system (FTC).
