# Supplementary material for: Cytometric patterns reveal growth states of Shewanella putrefaciens
Source: Microb Biotechnol. 2014 Sep 3;8(3):379–91. doi: 10.1111/1751-7915.12154 (PMC4408172; doi:10.1111/1751-7915.12154)
Supplement: Supplementary file 1 [file mbt20008-0379-sd1.doc]

**Supporting information**

Title: *Shewanella putrefaciens* proliferation dynamics in biofilms and planktonic cultures

Authors: Melzer S, Winter G, Jäger K, Hübschmann T, Hause G, Syrowatka F, Harms H, Tárnok A and Müller S

Corresponding author: susann.mueller@ufz.de

**Supplemental Experimental Procedures**

**Detailed description for cultivation of anaerobic biofilms on graphite paper**

Anaerobic biofilms were cultivated on rough graphite paper in a static chamber (250 ml Schott flask) under anaerobic conditions. The sealed anoxic chamber was filled with 200 ml lactate medium (lactate concentration of 2.67 g l^-1^, pH 7.4). Two notches plugged by a sterile septum were available for additional lactate medium supply and for waste drainage. After 46 h 2 ml of a lactate stock solution (1 g l^-1^ lactate in PBS, pH 7.2) and after 220 h of cultivation 5 ml lactate stock solution were supplemented as additional carbon and energy source. The graphite paper (Toray Carbon Paper TP-060, Quintech) had a size of 12.5 cm^2^ and served as electron acceptor for *S. putrefaciens* cells via connection to a second 250 ml Schott flask that was continuously aerated and contained a 10 cm^2^ Pt-cathode in 200 ml PBS, pH 7.2 (Toray Carbon Paper TP-060, 1 mg Pt/cm^2^, 10% hydrophobized, Quintech). Both graphite papers were connected by a copper clamp and through a 60 x 13 mm glass bride containing a Nafion® proton exchange membrane (117, DuPont), which was activated before use in 3 M H_2_SO_4_ for 1 h at RT. The assembly was similar to a simple microbial fuel cell design but current production was not measured in this study.

20 ml of a starter culture (from stationary growth phase, resuspended in PBS, pH 7.2) was inoculated and constantly stirred using a magnetic stirrer. A continuous nitrogen stream via the lid of the flask guaranteed anaerobic conditions. After 336 h the graphite paper grown cells were detached mechanically from the graphite paper by using a scalpel and 5 ml PBS (pH 7.2). The cell suspension was centrifuged at 3200*g and RT for 10 min. To determine the absolute number of detached cells 50 µl fluorescence beads (blue fluorescent beads: 1 μm, FluoSpheres 350/440, F-8815, Molecular Probes Eugene, Oregon, USA) were added to 450 µl DAPI stained detached biofilm cells and the resulting cell number was calculated to be 5.6 x 10^6^ cells/cm^2^. Graphite paper detached cells were fixated, stained, and measured as explained in the material and methods.

**Supporting figures**

**Cultivation parameters of *S. putrefaciens***

**
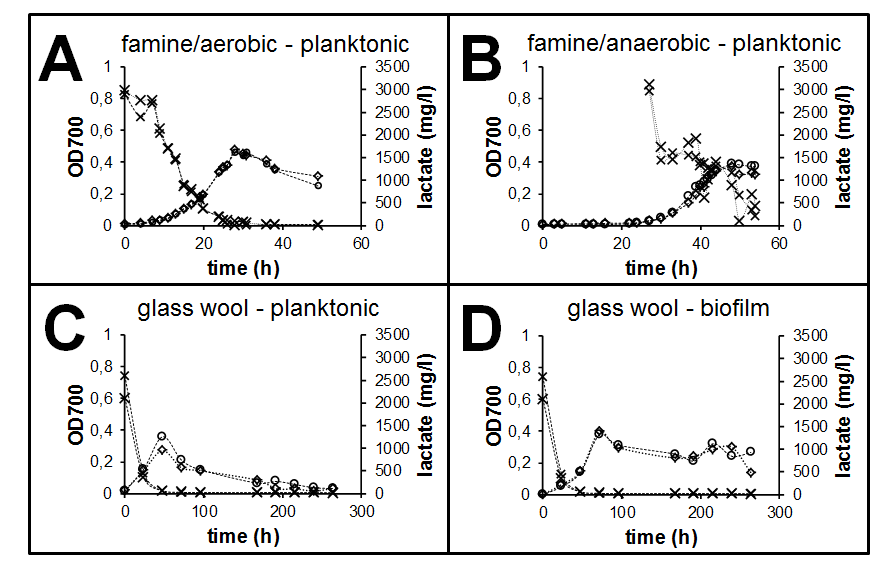
**

**Fig. S1**: **Substrate consumption of *S. putrefaciens*.** Lactate consumption was analyzed for growth under conditions A: lactate/aerobic (planktonic, in batch-culture), B: lactate+iron(III)citrate/anaerobic (planktonic, in batch-culture), C: lactate/aerobic (planktonic, in glass-wool approach), D: lactate/aerobic (biofilm, in glass-wool approach). Two independent measurements were performed.

**DNA subpopulations of *S. putrefaciens***

**Fig. S2:** **Gating strategy for** **differentiation of subpopulations with distinct DNA contents.** Signals by electronically noise were excluded in a DAPI:SSC gate (upper lane, left): all stained cells were gated (R1), as well as all events excluding the noise (R2). Total event count for unstained cells was calculated by subtraction of R1 from R2. Within gate R1 cells with C_1n_ (R3) to >C_6n_ (R9) were differentiated. Cultivating *S. putrefaciens* on glass wool resulted in an additional subpopulation with less than C_1n_ after 120 h (Fig. S8). Contamination was precluded due to colony isolation and identification via cultivation on plates. Geometric mean fluorescence values (MFI) for regions 3-9 for one (upper table) and for all measurements (average values, lower table) were calculated. The channel numbers were used for estimating chromosome equivalent numbers (C_n_). To set up heat-maps (Fig. 3 and Fig. S4) percentages for R3-R9 were calculated as shown in the formula (lower lane, left).

**Normalization of FCM data**

Changes in instrumental performance were recorded by daily measurement of a defined bacterial standard (*Acinetobacter sp.)* with constant DNA distribution and DAPI fluorescence intensity. For normalization measured relative changes in mean fluorescent intensity (rel. MFI) of the respective DNA subpopulation distributions over time were estimated and compared with the MFIs of the *S. putrefaciens* DNA patterns. The position of gates was corrected accordingly and enabled identification of C_1n_-C_4n_ states (Fig. S2).

**Fig. S3: Instrument stability over time**. A bacterial standard (*Acinetobacter* sp.) was measured each day by standardized preparation methods. The position of the first and the second peak distribution was evaluated by determination of the respective MFI values for every standard sample over 13 time points (corresponding to various *S. putrefaciens* cytometric measurements). The MFI values varied over time for distributions C_1n_ and C_2n_ due to instrument stability variations and were determined to be 60.25±13.8 and 116.26±25.5, respectively. The highest fluorescence intensity values were determined on day 11. These shifts were calculated and used for day specific correction for all DNA distribution data obtained during the various *S.* *putrefaciens* experiments (=data normalization).

**Fingerprint of DNA proliferation patterns of *S. putrefaciens***

**Fig. S4:** **Dynamics in bacterial growth pattern of planktonic and biofilm cells.** Distribution of DAPI stained cells with C_1n_ to > C_6n_ chromosome contents and less stained cells with < C_1n_ (LESS) were measured by FCM over cultivation time when planktonically grown under feast and aerobic (A), famine and aerobic (B), famine and anaerobic (C), and in matrix containing approaches (D). Data of biofilm grown cells are shown in (E). Time of cultivation is given in the first row of each heat-map. The color key marks abundance of cells in the < C_1n_ to > C_6n_ subpopulations in addition to unstained cells (abbreviated as UNS) from 0 – ≥70 % of all cells.

**Flow cytometric analysis of proliferation activity in *S. putrefaciens***


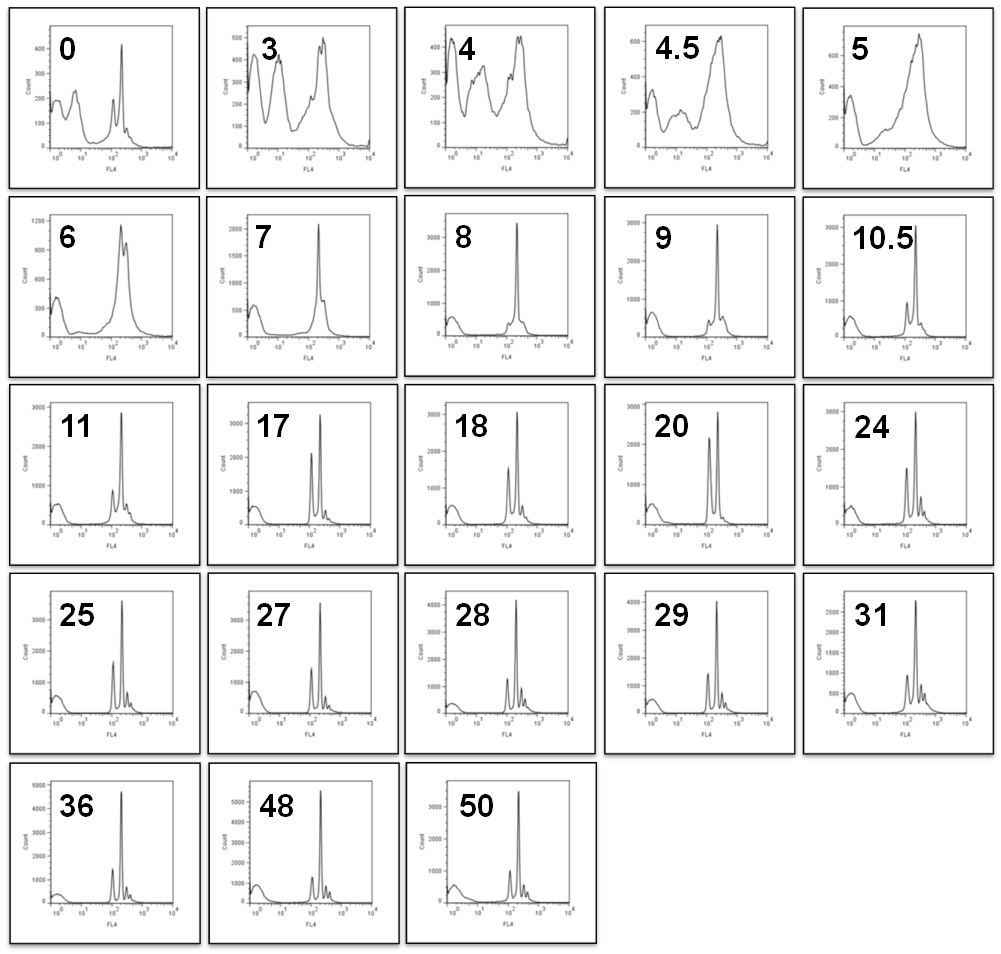


**Fig. S5:** **DNA-distribution patterns for feast growth under aerobic conditions.** Peptone was used as carbon source. During the first hours of cultivation (up to 5 hours) uncoupled DNA synthesis was clearly detected. Surprisingly, a high amount of cells remained DAPI unstained at the start of the batch cultivation (until 4.5 h). Frequencies of cells with one, two, three and four chromosome equivalents (C_1n_, C_2n_, C_3n_ and C_4n_) were quantified for each of the 23 time points.


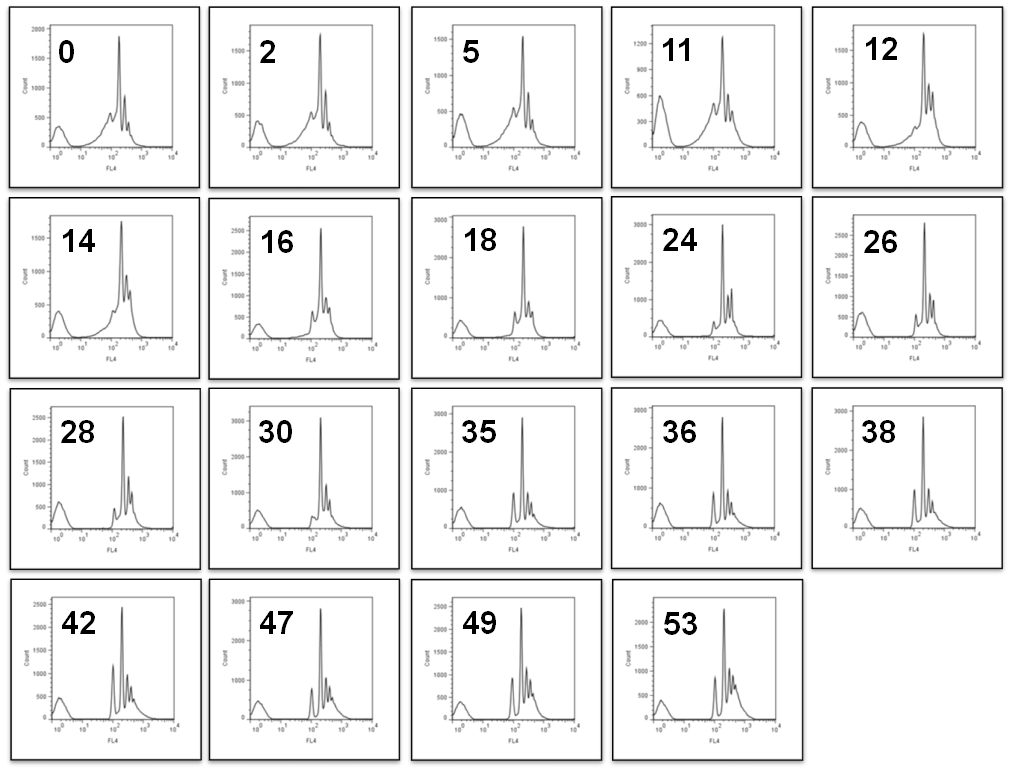


**Fig. S6:** **DNA-distribution patterns for famine growth under aerobic conditions.** Lactate was used as single carbon source. Samples were taken from 2 – 53 hours of cultivation to evaluate distribution of cells with C_1n_, C_2n_, C_3n_ and C_4n_.


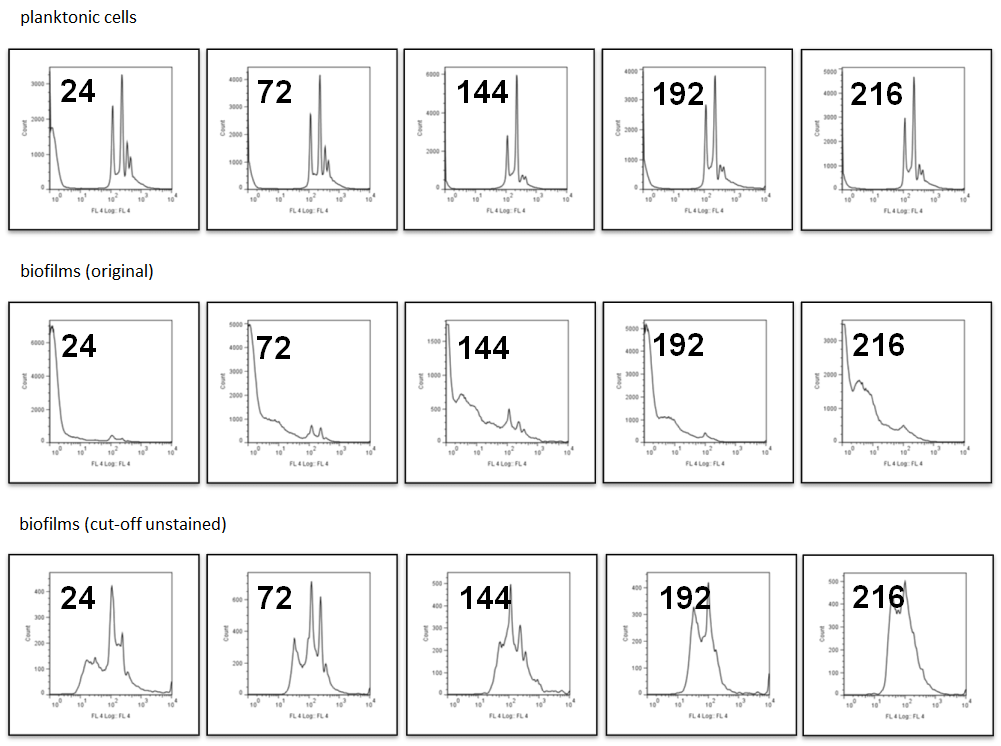


**Fig. S7:** **DNA-distribution pattern for planktonic and biofilm cells for the growth on glass slides.** Planktonic and biofilm cells from the flow-through chamber were analyzed at identical time points. Unstained cells were enriched in biofilm samples. To distinguish cells with C_1n_, C_2n_, C_3n_ and C_4n_ signal of unstained cells was excluded, resulting in a ‘cut-off unstained’ histogram.


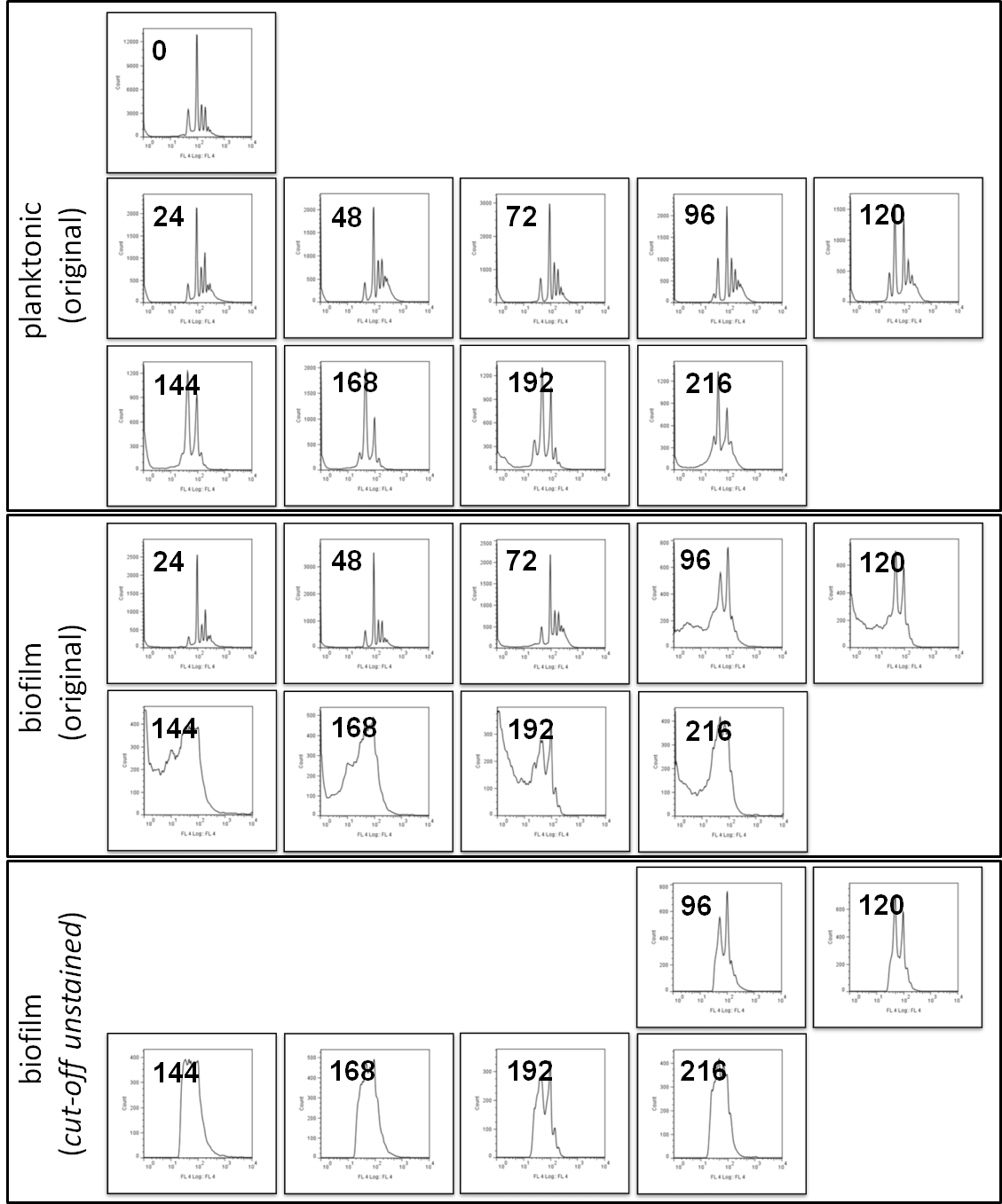


**Fig. S8:** **DNA-distribution pattern for planktonic and biofilm cells for the growth on glass wool.** Biofilm growth could be easily followed for early biofilms (up to 48 h). With prolonged incubation time unstained cells accumulated, resulting in difficulties to separate between cells with states of C_1n_, C_2n_, C_3n_ and C_4n_, therefore, unstained cells were excluded in ‘cut-off unstained histograms’, as described in Fig. S7. Cells with lowered stainability were found additionally in the planktonic cultures after 96 h of cultivation.


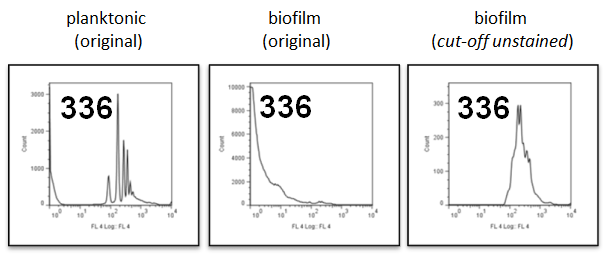


**Fig. S9:** **DNA-distribution pattern for planktonic and biofilm cells for growth on graphite paper**. Distribution of cells with C_1n_, C_2n_, C_3n_ and C_4n_ could be easily followed in planktonic samples, but was difficult to analyze for biofilm samples. Unstained cells were excluded in ‘cut-off unstained histograms’ which helped to differentiate between subsets with different DNA contents.


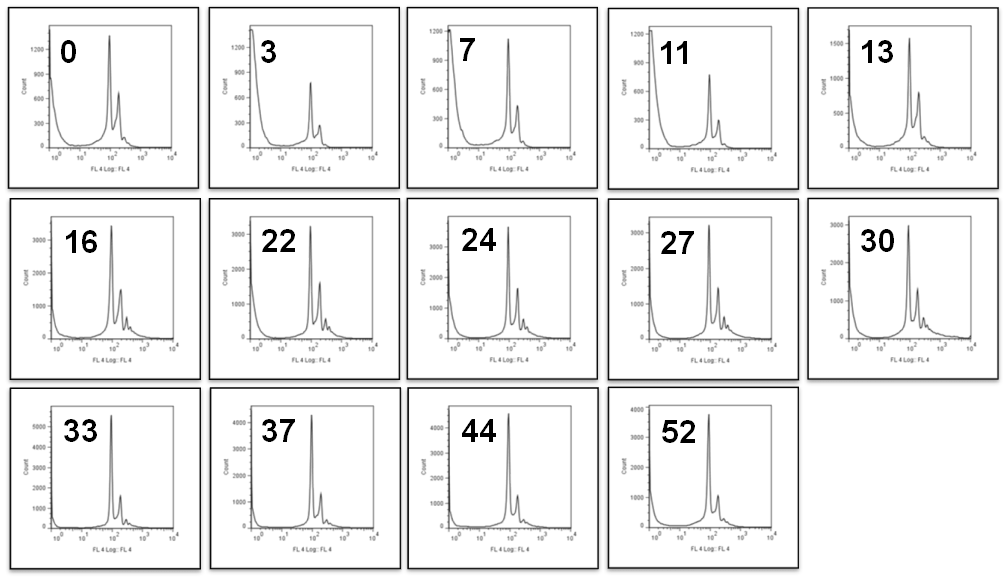


**Fig. S10:** **DNA-distribution pattern for famine growth under anaerobic conditions.** Lactate and iron(III)citrate were used as carbon source and electron acceptor, respectively. Distribution of cells with various chromosome equivalents was analyzed during growth for 52 hours.

**Dalmatian plot position correction**

A two parameter normalization approach was not available. However, in addition to the used DAPI fluorescent channel, also the scatter channels varied in their intensity recordings over time. Therefore, in two parameter measurements the position of subpopulations changed on the X-axis (side scatter) and Y-axis (DAPI fluorescence). To test, whether these changes influenced nMDS-plot analysis position-corrected vs. non-position-corrected test samples were compared with each other (Fig. S11). The results showed that position does not influence the comparison, in contrast to pattern distribution.


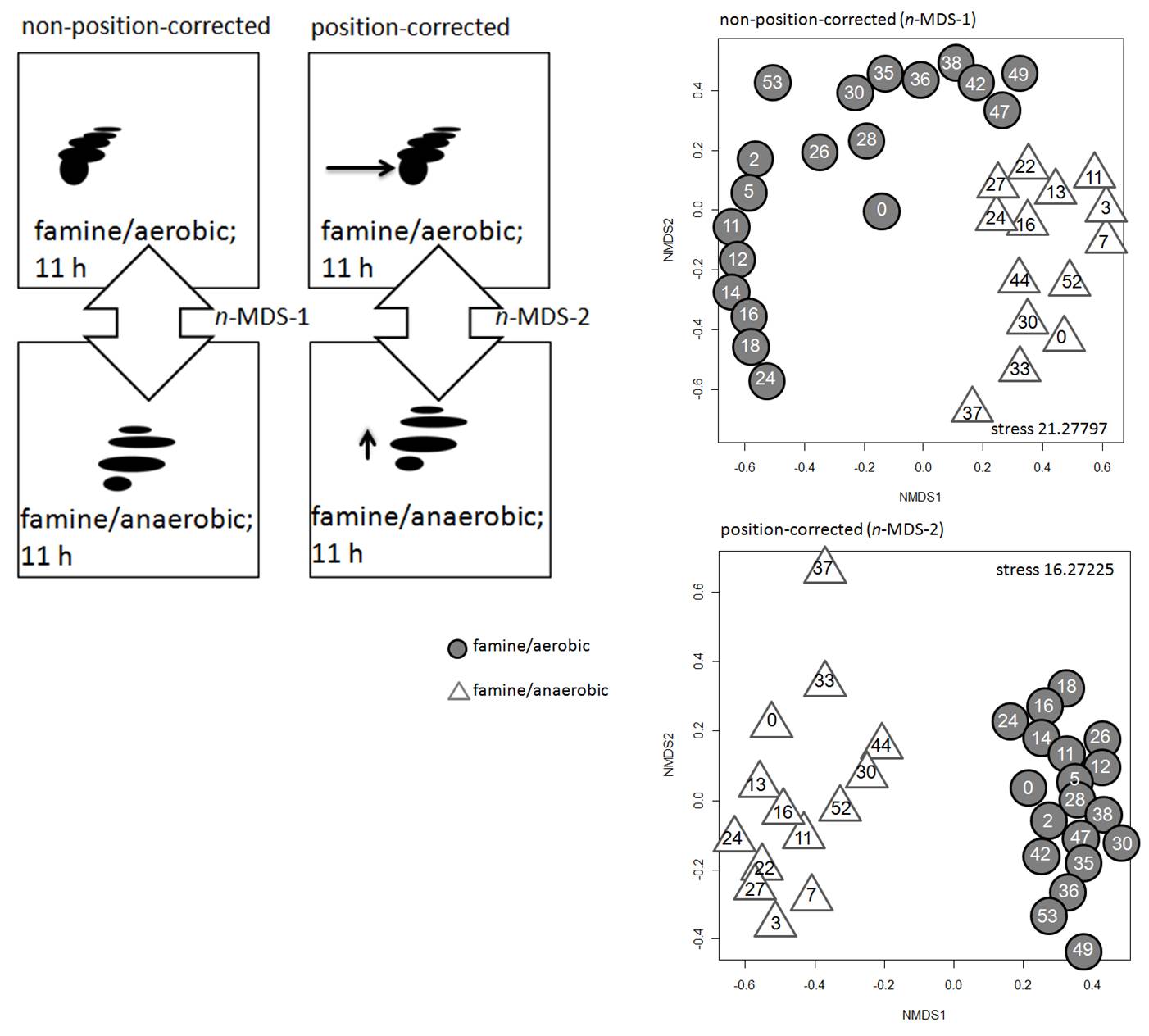


**Fig. S11:** **Dalmatian plot position tendency of comparative analysis via nMDS.** Position of the gates changed over time in the 2D plot on the x-axis (scatter) and y-axis (DAPI fluorescence). Data were therefore position corrected before nMDS analysis was performed. NMDS comparison was only slightly affected by population position, as shown by comparative nMDS analysis of position-corrected vs. non-position-corrected Dalmatian plots**.** Growth conditions could be clearly separated from each other with both data sets.

**Assembly of the Flow through chamber**

**Fig. S12: Schematic illustration of the flow-through-chamber system (FTC)**

Up to five glass slides were stacked into a Hellendahl staining chamber (1). Under aerobic conditions *S. putrefaciens* biofilms were cultivated on 2.67 g l^-1^ lactate. With a flow-rate of 4 ml/h by a peristaltic pump (4) fresh lactate medium (2) was added to the chamber constantly and waste was diverted (3). Samples were taken under sterile conditions for FCM and PC.
